# Supplementary material for: The ubiquitination landscape of the influenza A virus polymerase
Source: Nat Commun. 2023 Feb 11;14:787. doi: 10.1038/s41467-023-36389-0 (PMC9922279; doi:10.1038/s41467-023-36389-0)

Supplemental information:

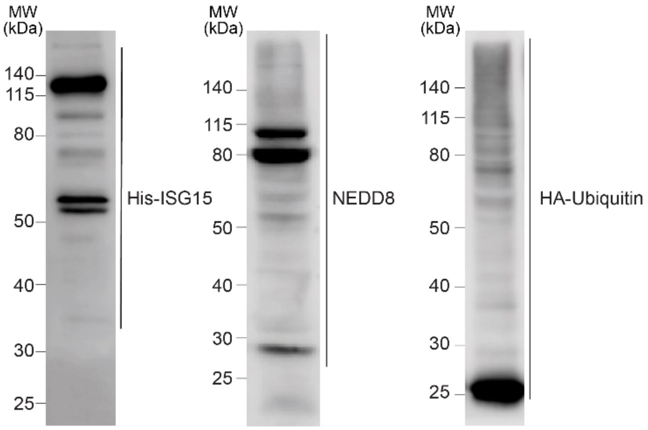

Supplementary Figure 1: Overexpression and immuno-detection of ubiquitin-like modifiers. HEK293-T cells were transfected with plasmids expressing His-tagged ISG15, HA-tagged NEDD8, HA-tagged ubiquitin. 24 h p.t., cells were lysed and analyzed by western blot. ISG15, NEDD8 and ubiquitin were detected using the indicated antibodies. Panels show representative blot of four independent experiments. MW, molecular weight marker.

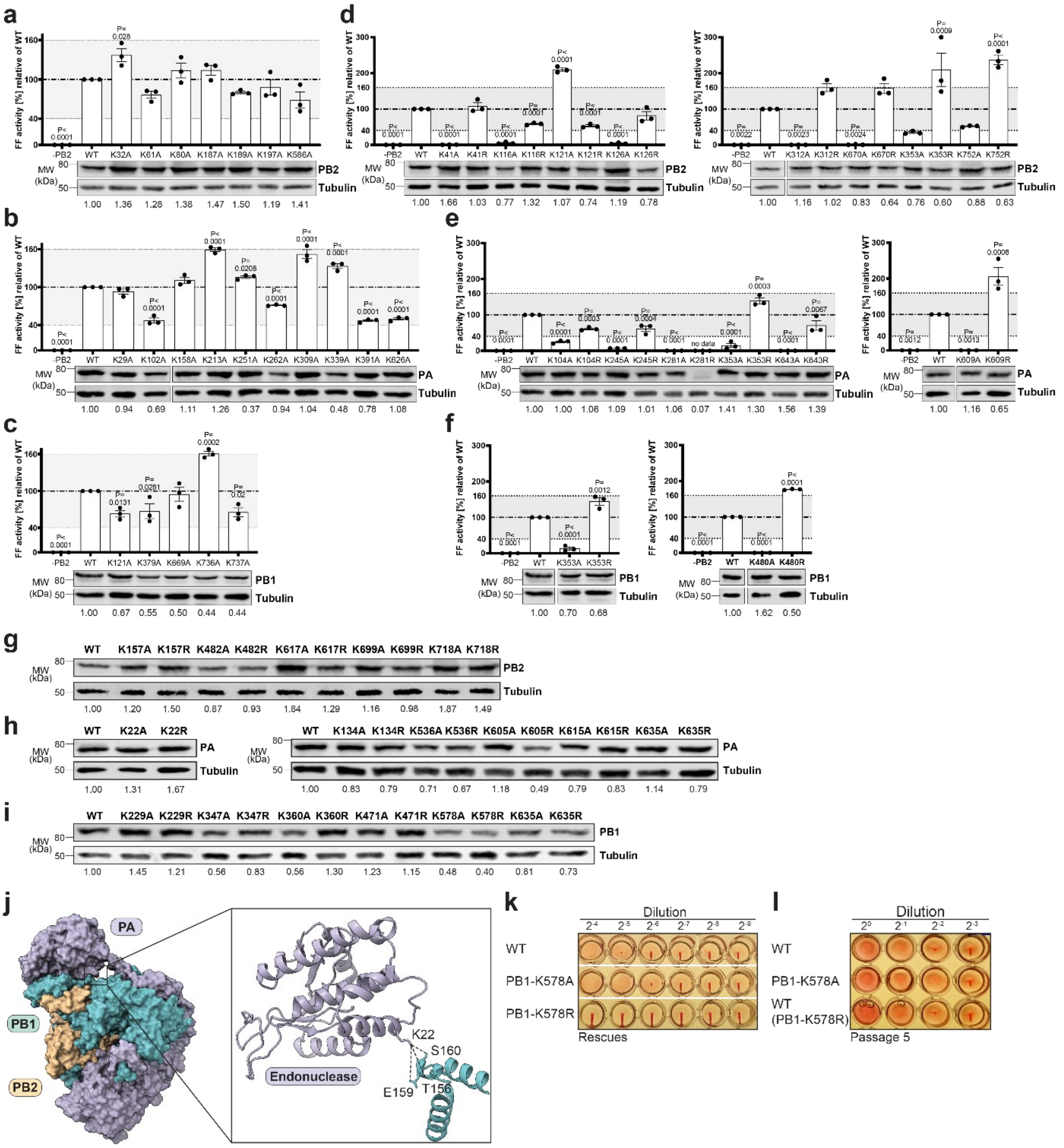

Supplementary Figure 2: Effects of lysine mutation to A and R on protein stability, polymerase activity and HI-titers.

a-f Polymerase reconstitution assay of the mutated polymerase complex with A/R-substitutions of the UB/UBL modified lysines in PB2 (a, d), PA (b, e) and PB1 (c, f). Depicted are positions showing polymerase activity within a cut-off of ± 60% wild type activity (indicated by grey boxes) upon A mutation and positions for which introduction of an arginine reverted the significant change in polymerase activity upon A mutation. Relative FF activities are reported as the mean percentage activity relative to WT (±SEM), n=3 independent biological replicates. P values <0.05 compared to WT from Dunnett's multiple comparison one-way ANOVA-test are indicated. For evaluating the expression levels of PB2, PB1 and PA, HEK293-T cells were transfected with plasmids encoding either wild type or mutant PB2, PB1 or PA. At 24 h p.t., expression levels were analyzed by western blot using the indicated antibodies. Representative blots from one experiment are shown. Quantified expression levels are reported below as relative values to WT. Tubulin expression served as the housekeeping control. **g-i** Expression of mutant PB2 (g), PA (h) and PB1 (i) was assessed as described above. **j** Illustration of the localization of PA-K22 in the 3D structures of the 1918 H1N1 polymerase heterotrimer (PDB: 7NHA; Supplementary Table 1) showing the K22 containing hinge region of the PA endonuclease domain (violet) and the interacting sites in the PB1 subunit (cyan). Created with ChimeraX. **k-l** Determination of the titers of

recombinant viruses using the hemagglutination inhibition (HI) assay. Virus rescue was carried out using the pHW2000 plasmid system with either wild type or mutant PB1. 48 h p.t., HI titers in the supernatants after rescue (k) were evaluated using the same volume of undiluted supernatant. Viruses were propagated on A549 cells for 5 passages (MOI: 0.01, except for the first passage of PB1-K578R: MOI=0.001) and viral titers were examined 48 h. p.i. HI titers at passage 5 (l) were determined by using 10^6 PFU. PB1-K578R was classified as reversion to WT based on sequencing analyses. Source data are provided as a Source data file.

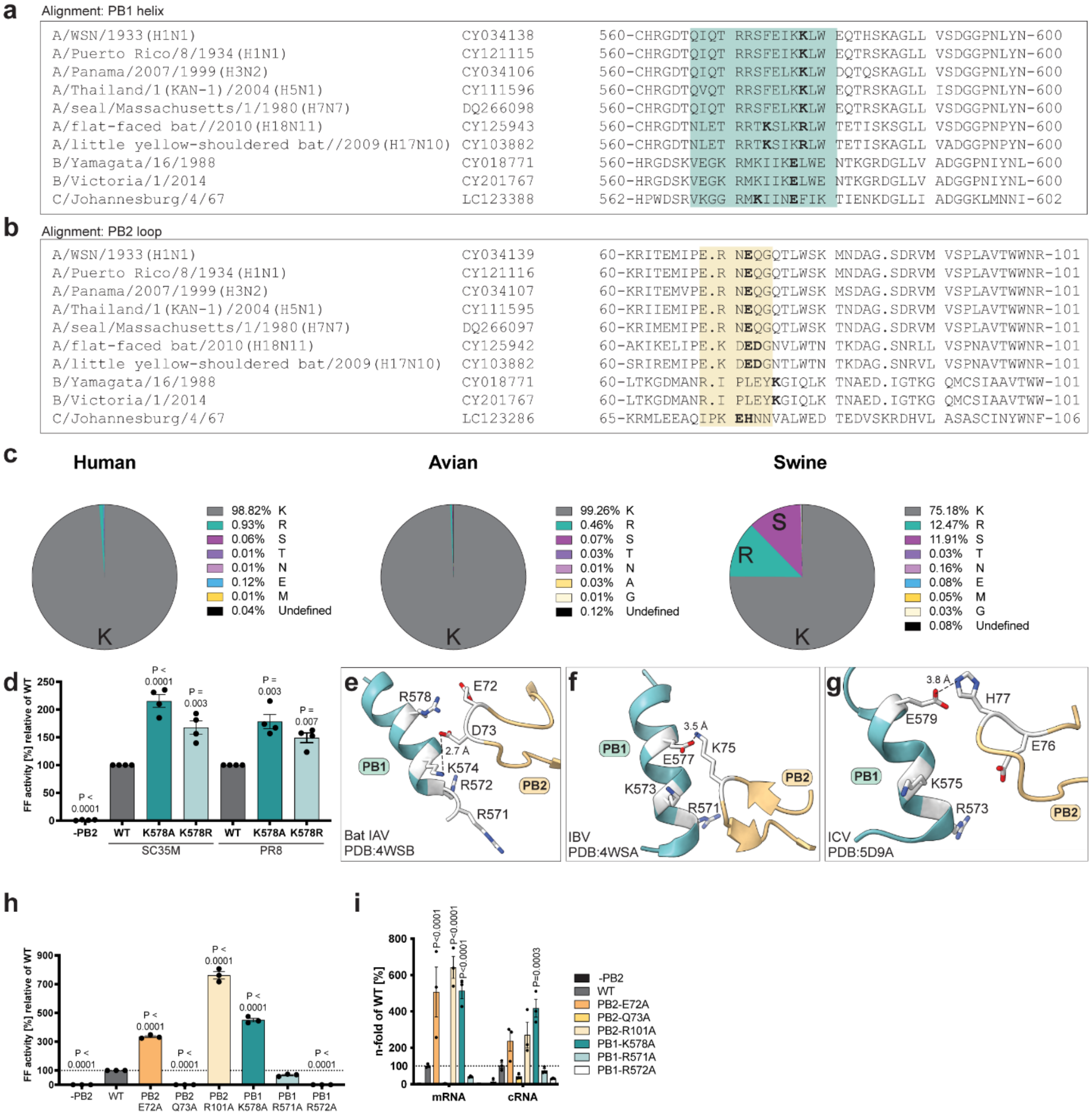

Supplementary Figure 3: Conservation and structural environment of PB1-K578 and PB2-E72 in different influenza viruses.

a-b Sequence alignment of PB1 AA 560-600 and PB2 AA 60-101. The PB1-K578 helix is highlighted in cyan (a), the N-terminal PB2 loop is highlighted in yellow (b) in the indicated strains. **c** Conservation of PB1-K578 and the relative distribution of alternative amino acids in human (left panel), avian (middle panel) and swine (right panel) isolates (sequences downloaded from NCBI (PB2 and PB1: 09/15/2017, PA: 10/01/2017) presented as percentages of all analyzed sequences. **d** Polymerase activity of PB1-K578A/R mutants in the polymerase reconstitution assay of IAV strains SC35M and PR8. Relative FF activities are presented as the mean percentage activity relative to SC53M or PR8 WT (±SEM), n=4 independent biological replicates. P values <0.05 compared to WT from Dunnett's multiple comparison one-way ANOVA-test are indicated. **e-g** Zoom-in into 3D structures of bat Influenza virus A (e; PDB: 4WSB), Influenza virus B (f; PDB:4WSA) and Influenza virus C (g; PDB: 5D9A) and illustration of interacting amino acids located in the interface that covers the PB1-578 containing helix (cyan) and the N-terminal PB2 loop (yellow). Distances between electrostatically interacting amino acids that reside in the interface are indicated in Ångstrom (Å). Created with ChimeraX. **h** Polymerase reconstitution assay of the mutated polymerase complex with A-substitutions in the positively charged interaction surface and the PB2 loop. Relative FF activities are presented as the mean percentage activity relative to WT (±SEM), n=3 independent biological replicates. P values <0.05 compared to WT from Dunnett's multiple comparison one-way ANOVA-test are indicated. **i** Quantification of FF mRNA and cRNA levels from the

polymerase reconstitution assay depending on the indicated mutations in PB2 and PB1 using qRT-PCR. mRNA and cRNA levels were detected using specific primers and are depicted as mean relative to the WT polymerase (±SEM), n=3 independent biological replicates. Cellular *GAPDH* mRNA levels were used as housekeeping control. P values <0.05 compared to WT from Dunnett's multiple comparison two-way ANOVA test are indicated. Source data are provided as a Source data file.

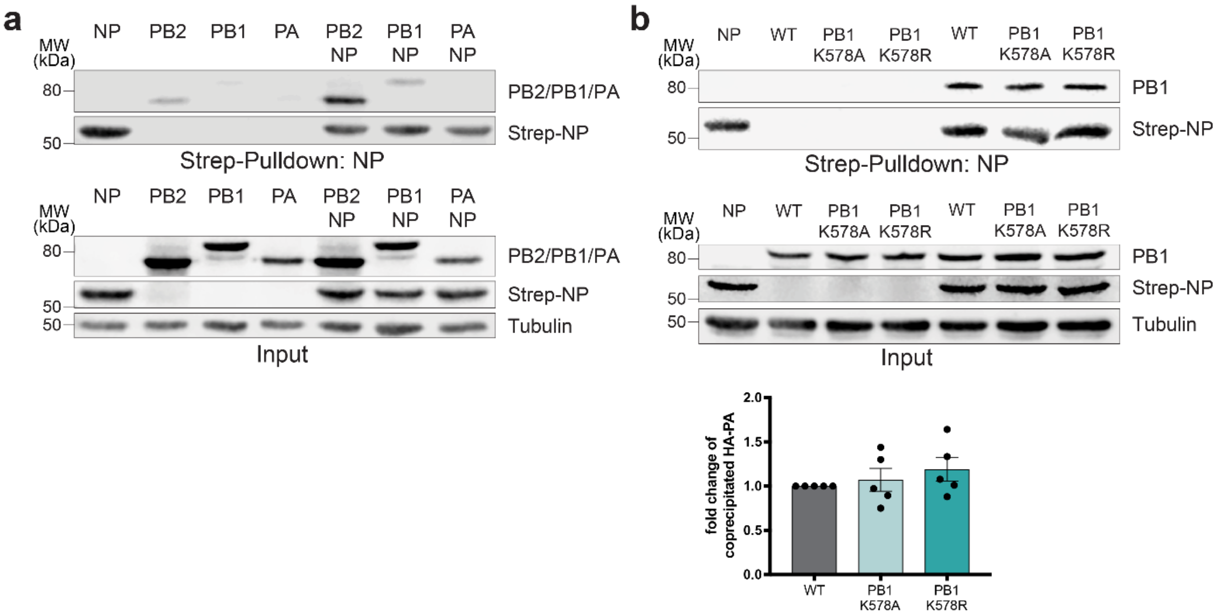

Supplementary Figure 4:

a Assessment of NP binding to the individual polymerase subunits PB2, PB1 and PA using co-affinity precipitation. HEK293-T cells were transfected with wild type PB1, PB2, PA together with or without strep-tagged NP, respectively. At 24 h p.t. polymerase subunits bound to NP were strep-purified and analyzed by western blot using the indicated antibodies. A representative blot from three independent experiments is shown. **b** Assessment of NP binding to PB1 using co-affinity precipitation. HEK293-T cells were transfected with wild type PB1 (WT) or mutant PB1-K578A/R with or without strep-tagged NP, respectively. At 24 h p.t. PB1 bound to NP was strep-purified and analyzed by western blot. Quantification of the interaction is shown below. Levels of PB1 were normalized to strep-tagged NP and reported below as the mean n-fold of WT (±SEM), n=5 independent biological replicates. Dunnett's multiple comparison one-way ANOVA test showed no significant difference compared to WT (p>0.05). Source data are provided as a Source data file.

Supplementary Table 1: Modified sites with charge-dependent effects on the RdRP activity: List of interacting residues

Site	Polar interaction	Structure	PDB
		**PB2**	
K41	vRNA	Transcription (Bat IAV):	
		Pre-initiation complex	6T0N
		Termination complex	6TW1
		Stuttering complex	6T0S
	PB1-Y30	Elongation complex	6T0V
K116	PB2-E120	Model: WSN polymerase (vRNA bound)	
		Transcription (Bat IAV):	
		Pre-initiation complex	6T0N
		Model: WSN polymerase (cRNA bound)	
K121	PB1-H605	Model: WSN polymerase (vRNA-bound)	
		Transcription (Bat IAV):	
		Pre-initiation complex	6T0N
		Elongation complex	6T0V
		Pre-Termination complex	6SZU
		Termination complex	6TW1
		Recycling complex	6T2C
	PB1-E656	Pre-Initiation complex	6T0N
		Asymmetric dimer (both polymerases) [9]	
K126	PB1-E614	Symmetric dimer	6QNW
	PA-D431	Asymmetric dimer (both polymerases) [9]	
	PB1-R602	Asymmetric dimer (both polymerases) [9]	
K312	PB2-E304	Model: WSN polymerase (cRNA bound)	
	PB2-L298		
	PB2-N301	Model: WSN polymerase (vRNA bound)	
	PB2-T303		
K353	PB2-E341	Cap-binding domain with cap	2VQZ
K670	PB2-Q136	Model: WSN polymerase (vRNA bound)	
		Transcription (Bat IAV):	
		Elongation complex	6T0V
		Termination complex	6TW1
	PB2-E681	Model: WSN polymerase (cRNA bound)	
		**PB1**	
K353	vRNA	Transcription (Bat IAV):	
		Elongation complex	6T0V
		Pre-Termination complex	6SZU
		Termination complex	6TW1
		**PA**	
K22	PB1-T156	1918 H1N1 polymerase heterotrimer	7NHA
	PB1-E159		
	PB1-S160		
K104	mRNA?	Model: PA_N–RNA complex [50]	
K245	PB1-Q84 and	Model: WSN polymerase (vRNA bound)	
	PB1-D86	Transcription (Bat IAV):	
		Pre-initiation complex	6T0N
		Elongation complex	6T0V
		Pre-Termination complex	6SZU
		Stuttering complex	6T0S
		Dissociation complex	6T0U
		Recycling complex	6T2C
K353	PA-K353	Symmetric dimer interface	6QNW
K609	PA-D383	Asymmetric dimer interface [9]	
K643	PB1-T20	Model: WSN polymerase (vRNA bound)	
		Symmetric dimer	6QNW
		Asymmetric dimer: encapsidating Pol [9]	
	PB1-E491	Asymmetric dimer: replicating Pol [9]	

Supplementary Table 2: p values generated by Welch corrected t-test for RMSF values

PB2 residue		WT / K578R	WT / K578A	K578R / K578A
61	K	0.8316	0.0011	0.0003
62	R	0.7189	0.0042	0.0009
63	I	0.5804	0.0091	0.0012
64	T	0.4036	0.0242	0.0014
65	E	0.4231	0.0659	0.0066
66	M	0.2344	0.2657	0.0165
67	I	0.1385	0.3526	0.0109
68	P	0.2646	0.4987	0.0475
69	E	0.3717	0.4090	0.0558
70	R	0.4684	0.5967	0.1590
71	N	0.3053	0.5821	0.0740
72	E	0.2752	0.7741	0.1233
73	Q	0.2785	0.9215	0.2695
74	G	0.3277	0.6267	0.5316
75	Q	0.3282	0.4267	0.8021
76	T	0.4744	0.5124	0.9243
77	L	0.5049	0.4525	0.9313
78	W	0.5201	0.3883	0.7857
79	S	0.5539	0.3923	0.7229
80	K	0.7218	0.4878	0.5444
81	M	0.7957	0.7996	0.3868
82	N	0.2275	0.6958	0.0346

Uncropped Blots from supplementary data:

Supplementary Figure 2

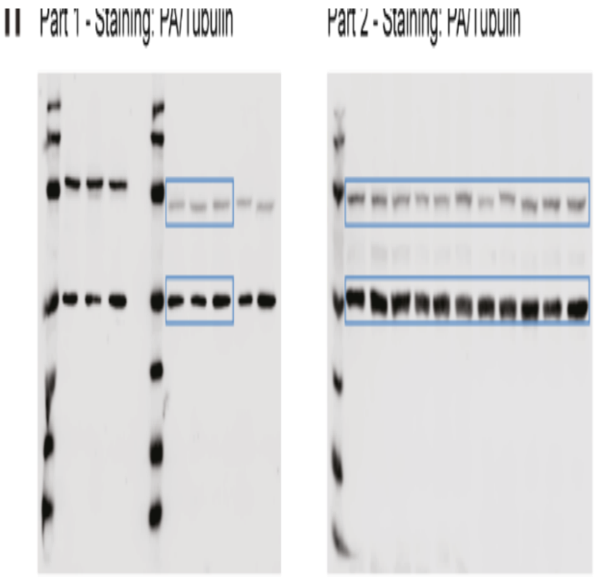

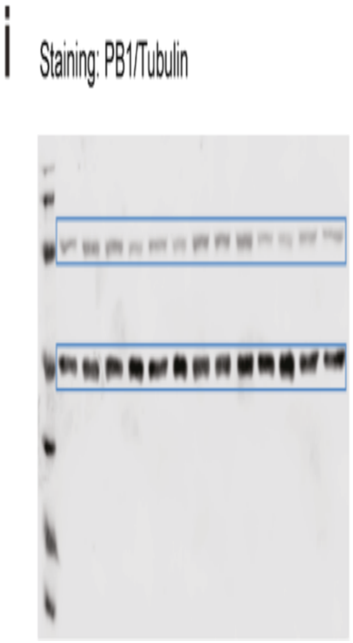

Supplementary Figure 4

a Pulldown - Staining: PB2/PB1/PA/NP   Input - Staining: PB2/PB1/PA/NP/Tubulin

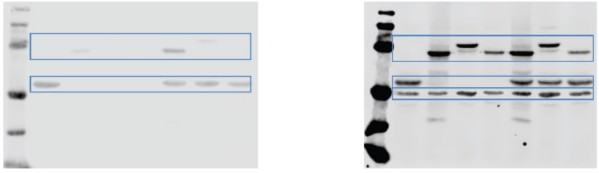

b Pulldown - Staining: PB2/NP   Input - Staining: PB1   NP   Tubulin

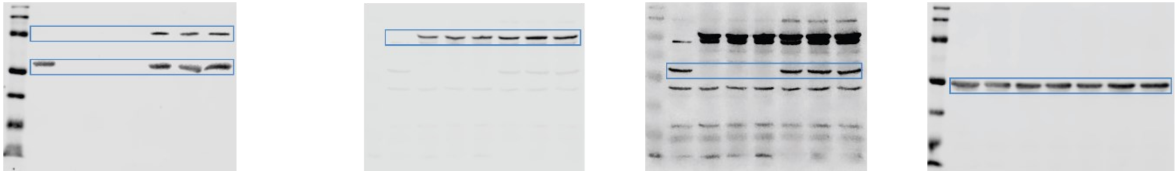

Supplement: Supplementary file 1 — Supplementary Information [file 41467_2023_36389_MOESM1_ESM.pdf]
