# Supplementary material for: The ubiquitination landscape of the influenza A virus polymerase
Source: Nat Commun. 2023 Feb 11;14:787. doi: 10.1038/s41467-023-36389-0 (PMC9922279; doi:10.1038/s41467-023-36389-0)
Supplement: Supplementary file 3 — Description of Additional Supplementary Files [file 41467_2023_36389_MOESM3_ESM.docx]

**Description of additional supplementary files**

Title: Supplementary data 1

Description: This file contains information on primers sequences used in this study.
