# Supplementary material for: The ubiquitination landscape of the influenza A virus polymerase
Source: Nat Commun. 2023 Feb 11;14:787. doi: 10.1038/s41467-023-36389-0 (PMC9922279; doi:10.1038/s41467-023-36389-0)
Supplement: Supplementary file 5 — Reporting Summary [file 41467_2023_36389_MOESM5_ESM.pdf]

nature portfolio

Corresponding author(s): Linda Brunotte
Last updated by author(s): Jan 17, 2023

Reporting Summary

Nature Portfolio wishes to improve the reproducibility of the work that we publish. This form provides structure for consistency and transparency in reporting. For further information on Nature Portfolio policies, see our Editorial Policies and the Editorial Policy Checklist.

Statistics

For all statistical analyses, confirm that the following items are present in the figure legend, table legend, main text, or Methods section.

n/a	Confirmed	
☐	☒	The exact sample size (*n*) for each experimental group/condition, given as a discrete number and unit of measurement
☐	☒	A statement on whether measurements were taken from distinct samples or whether the same sample was measured repeatedly
☐	☒	The statistical test(s) used AND whether they are one- or two-sided<br>*Only common tests should be described solely by name; describe more complex techniques in the Methods section.*
☒	☐	A description of all covariates tested
☒	☐	A description of any assumptions or corrections, such as tests of normality and adjustment for multiple comparisons
☐	☒	A full description of the statistical parameters including central tendency (e.g. means) or other basic estimates (e.g. regression coefficient) AND variation (e.g. standard deviation) or associated estimates of uncertainty (e.g. confidence intervals)
☐	☒	For null hypothesis testing, the test statistic (e.g. *F*, *t*, *r*) with confidence intervals, effect sizes, degrees of freedom and *P* value noted<br>*Give P values as exact values whenever suitable.*
☒	☐	For Bayesian analysis, information on the choice of priors and Markov chain Monte Carlo settings
☒	☐	For hierarchical and complex designs, identification of the appropriate level for tests and full reporting of outcomes
☒	☐	Estimates of effect sizes (e.g. Cohen's *d*, Pearson's *r*), indicating how they were calculated

Our web collection on statistics for biologists contains articles on many of the points above.

Software and code

Policy information about availability of computer code

Data collection	LI-COR Image Studio™ (v5.2.5), Visual Molecular Dynamics (VMD; v1.9.3), ZEN (v2.6), MODELLER (v9.19); YASARA structure (v21.6.2 and v21.8.27), LightCycler 480 Gene Scanning Software, LI-COR Image Studio™ (v5.2.5)
Data analysis	ChimeraX (v1.3.), PyMOL Molecular Graphics System (v2.3.0), GraphPad PRISM™ (v7.04), Origin 2022 (v9.9), Ridom SeqSphere+ software (v7), Visual Molecular Dynamics (VMD; v1.9.3), Yasara structure (v21.6.2 and v21.8.27), ZEN (v2.6), MaxQuant (v. 2.0.1.0), ImageJ (v1.53c), LI-COR Image Studio™ (v5.2.5)

For manuscripts utilizing custom algorithms or software that are central to the research but not yet described in published literature, software must be made available to editors and reviewers. We strongly encourage code deposition in a community repository (e.g. GitHub). See the Nature Portfolio guidelines for submitting code & software for further information.

Data

Policy information about availability of data

All manuscripts must include a data availability statement. This statement should provide the following information, where applicable:

- Accession codes, unique identifiers, or web links for publicly available datasets
- A description of any restrictions on data availability
- For clinical datasets or third party data, please ensure that the statement adheres to our policy

The mass spectrometry proteomics data generated in this study have been deposited to the ProteomeXchange Consortium via the PRIDE partner repository with

the dataset identifier PXD030816 and 10.6019/PXD030816. Username and password are available upon reasonable request.
MD simulation data are available upon reasonable request.
The study made use of the publicly available datasets:
NCBI: PB1, PB2 and PA sequences from isolates of human, swine and avian IAV origin. Downloaded: PB2 and PB1: 09/15/2017. PA: 10/01/2017.
PDB entries: 4WSB, 5EPI, 6QNW, 7NHA, 4WSA, 5D9A, 6T0N, 6TW1, 6T0S, 6T0V, 6SZU, 6T2C, 6T0U, 2VQZ
GenBank: accession numbers CY034132-CY034139
UniprotKB database (UP000000834.fasta version from 10/2016; UP000005640_9606.fasta version from 04/2019).
Biochemical data generated in this study are provided in the Source data file.

Human research participants

Policy information about studies involving human research participants and Sex and Gender in Research.

Reporting on sex and gender	n/a
Population characteristics	n/a
Recruitment	n/a
Ethics oversight	n/a

Note that full information on the approval of the study protocol must also be provided in the manuscript.

Field-specific reporting

Please select the one below that is the best fit for your research. If you are not sure, read the appropriate sections before making your selection.

☒ Life sciences ☐ Behavioural & social sciences ☐ Ecological, evolutionary & environmental sciences

For a reference copy of the document with all sections, see nature.com/documents/nr-reporting-summary-flat.pdf

Life sciences study design

All studies must disclose on these points even when the disclosure is negative.

Sample size	Sample size were estimated on the basis of previous studies using similar methods, see Chen et al. (DOI:10.1371/journal.ppat.1008034); Giese et al. (DOI: 10.1038/s41467-017-01112-3); Zhang et al. (DOI: 10.1038/srep43691)
Data exclusions	No data were excluded.
Replication	Biochemical experiments were performed with at least three independent replicates (unless otherwise stated), with all attempts at replication successfull. In Figure 5g one replicate of NA vRNA levels for WT could not be determined. Immune fluorescence analyses of A549 cells overexpressed with plasmids encoding for WT or mutant PB2, PB1 and PA was performed once (Figure 3 l-n). Analysis of protein expression of plasmids encoding for WT or mutant PB2, PB1 and PA was performed once (Supplementary Fig. 2 a-i).
Randomization	Randomization is not relevant to our study, given that samples were not assigned to any groups.
Blinding	Blinding was not relevant for our study. since there are no experiments where investigator bias could affect measurements or data analysis. All data were analyzed using unbiased methods.

Reporting for specific materials, systems and methods

We require information from authors about some types of materials, experimental systems and methods used in many studies. Here, indicate whether each material, system or method listed is relevant to your study. If you are not sure if a list item applies to your research, read the appropriate section before selecting a response.

Materials & experimental systems

n/a	Involved in the study
☐	☒ Antibodies
☐	☒ Eukaryotic cell lines
☒	☐ Palaeontology and archaeology
☒	☐ Animals and other organisms
☒	☐ Clinical data
☒	☐ Dual use research of concern

Methods

n/a	Involved in the study
☒	☐ ChIP-seq
☒	☐ Flow cytometry
☒	☐ MRI-based neuroimaging

Antibodies

Antibodies used

Rat anti-HA-Tag (clone 3F10, Roche, Germany), Rabbit anti-NEDD8 (clone Y297, Abcam, UK), Mouse anti-His-Tag (clone HIS.H8, Thermo Fisher, USA), Rabbit anti-PB2 (GTX125926, Genetex, USA), Rabbit anti-PB1 (GTX125923, Genetex, USA), Rabbit anti-PA (GTX125932, Genetex, USA), Rabbit anti-NP (GTX125989, Genetex, USA), Mouse anti-Strep-Tag (clone GT661, Sigma Aldrich, Germany), Mouse anti-Tubulin (clone DM1A, Sigma Aldrich, Germany),

anti-Mouse IgG-680RD (LI-COR, Bad Homburg, Germany), anti-Mouse IgG - HRP (Cell signaling Technologies, Frankfurt, Germany), anti-Rabbit IgG-680RD (LI-COR, Bad Homburg, Germany), anti-Rabbit IgG-800CW (LI-COR, Bad Homburg, Germany), anti-Rabbit IgG - HRP (Cell signaling Technologies, Frankfurt, Germany), anti-Rabbit IgG-Alexa Flour 488 (Thermo Fisher Scientific, Schwerte, Germany), anti-Rabbit IgG-Alexa Flour 568 (Thermo Fisher Scientific, Schwerte, Germany), anti-Rat IgG-800CW (LI-COR, Bad Homburg, Germany), anti-Rat IgG-HRP (Cell signaling Technologies, Frankfurt, Germany), anti-Rat IgG-Alexa Fluor 488 (Thermo Fisher Scientific, Schwerte, Germany)

Validation

Commercially available antibodies were all validated by suppliers:
Rat anti HA-Tag (clone 3F10, Roche) https://www.sigmaaldrich.com/DE/en/product/roche/12158167001
Rabbit anti-NEDD8 (clone Y297, Abcam) https://www.abcam.com/nedd8-antibody-y297-ab81264.html?
Mouse anti-His-Tag (clone HIS.H8, Thermo Fisher) https://www.thermofisher.com/antibody/product/6x-His-Tag-Antibody-clone-HIS-H8-Monoclonal/MA1-21315
Rabbit anti-PB2 (GTX125926, Genetex) https://www.genetex.com/Product/Detail/Influenza-A-virus-PB2-protein-antibody/GTX125926
Rabbit anti-PB1 (GTX125923, Genetex) https://www.genetex.com/Product/Detail/Influenza-A-virus-PB1-protein-antibody/GTX125923
Rabbit anti-PA (GTX125932, Genetex) https://www.genetex.com/Product/Detail/Influenza-A-virus-PA-protein-antibody/GTX125932
Rabbit anti-NP (GTX125989, Genetex) https://www.genetex.com/Product/Detail/Influenza-A-virus-Nucleoprotein-antibody/GTX125989
Mouse anti-Strep-Tag (clone GT661, Sigma Aldrich) https://www.sigmaaldrich.com/DE/en/product/sigma/sab2702216
Mouse anti-Tubulin (clone DM1A, Sigma Aldrich) https://www.sigmaaldrich.com/DE/en/product/mm/mabt205

Eukaryotic cell lines

Policy information about cell lines and Sex and Gender in Research

Cell line source(s)

A549: ATCC; # CCL-185; MDCK II: Merck; #00062107; HEK293T cells: ATTC; CRL-3216. All cells were kindly provided by Prof. Martin Schwemmle (Institute of Virology, Freiburg, Germany)

Authentication

Cell lines were not authenticated.

Mycoplasma contamination

All cells were tested negative for mycoplasma contamination.

Commonly misidentified lines
(See ICLAC register)

No commonly misidentified cell lines were used.
